# Oral bioavailability enhancement of doxazosin mesylate: Nanosuspension versus self-nanoemulsifying drug delivery systems

**DOI:** 10.5599/admet.2022

**Published:** 2023-11-03

**Authors:** Al Zahraa G. Al Ashmawy, Mohammad H. Alyami, Noura G. Eissa, Gehan F. Balata, Hanan M. El Nahas

**Affiliations:** 1 Department of Pharmaceutics and Pharmaceutical Technology, Faculty of Pharmacy, Heliopolis University, Cairo 11785, Egypt; 2 Department of Pharmaceutics, College of Pharmacy, Najran University, Najran 66462, Saudi Arabia; 3 Badr University in Cairo Research Center, Badr University in Cairo, Badr City, Cairo 11829, Egypt; 4 Department of Pharmaceutics and Industrial Pharmacy, Faculty of Pharmacy, Zagazig University, Zagazig 44519, Egypt; 5 Pharmacy Practice Department, Faculty of Pharmacy, Heliopolis University, Cairo 11785, Egypt

**Keywords:** hypertension, solubility, zeta potential, mean arterial blood pressure, dissolution

## Abstract

**Background and purpose:**

Doxazosin mesylate (DOX) is an antihypertensive drug that possesses poor water solubility and, hence, poor release properties. Both nanosuspensions and self-nanoemulsifying drug delivery systems (SNEDDS) are becoming nanotechnology techniques for the enhancement of water solubility of different drugs.

**Experimental approach:**

The study's goal was to identify the best drug delivery system able to enhance the release and antihypertensive effect of DOX by comparing the physical characteristics such as particle size, zeta potential, entrapment efficiency, release rate, and main arterial blood pressure of DOX-loaded nanosuspensions and SNEDDS in liquid and solid form.

**Key results:**

DOX nanosuspension preparation had a particle size of 385±13 nm, poly-dispersity index of 0.049±3, zeta potential of 50 ± 4 mV and drug release after 20 min (91±0.43 %). Liquid SNEDDS had a droplet size of 224±15 nm, poly-dispersity index of (0.470±0.01), zeta potential of -5±0.10 mV and DR20min of 93±4 %. Solid SEDDS showed particle size of 79±14 nm, poly-dispersity index of 1±0.00, a zeta potential of -18 ±0.26 mv and DR20min of 100 ±2.72 %.

**Conclusion:**

Finally, in terms of the mean arterial blood pressure lowering, solid SNEDDS performed better effect than both liquid SNEDDS and nanosuspension (*P* >0.05).

## Introduction

There are many different pharmacological compounds and drug candidates in the world of pharmaceutical product development, however, despite their therapeutic efficacy, they frequently have poor water solubility, so the rate of drug release and absorption will be limited, reflecting on the therapeutic drug effect. For improving the low solubility and release rate of hydrophobic drugs, many strategies, including solid dispersion [1], micronization [2], pH modification [3], crystal modification [4], and self-emulsifying drug delivery systems, have been investigated [5]. Both nanosuspensions and self-nanoemulsifying drug delivery systems (SNEDDS) are emerging nanotechnology techniques for the enhancement of water solubility and bioavailability of poorly water-soluble drugs with safe components [6,7]. Nanosuspensions are submicron colloidal dispersions of drugs stabilized by surfactants [7]. The drug powder is transferred to drug nanoparticles using bottom-up and top-down technologies [8]. Nanosuspension emerged as a solution to deliver hydrophobic drugs. Scaling down nanoparticles increases surface area and enhances drug aqueous solubility and bioavailability [8,9]. The main components of nanosuspensions are stabilizers, organic solvents, surfactants, co-surfactants and cryoprotectants [10].

SNEDDS are anhydrous mixtures of oil, surfactant and co-surfactant in which the drug is dissolved. According to their capacity to spontaneously generate nanoemulsion in the gastrointestinal environment following oral administration, suitable excipients for SNEDDS, such as oil, surfactant, and co-surfactant, are chosen [11,12]. Additionally, in situ solubilized drugs that develop in the gastrointestinal tract (GIT) lumen can be ultimately absorbed via the lymphatic system without going through the liver's first-pass metabolism [13]. They are kept anhydrous until diluted with gastrointestinal tract fluids and turned into oil in water emulsion of very small size, nearly 200 nm or less [6,14]. SNEDDS become preferable to nanoemulsions for many reasons, including that SNEDDS give rise to more stable formulations that can be stored longer. Also, it can be easily filled into capsules [6]. Both nanosuspensions and nanoemulsions are able to increase water solubility and, hence, bioavailability of poorly water-soluble drugs.

Doxazosin mesylate (DOX), α1-adrenergic receptor blocker, is an antihypertensive drug that suffers from low bioavailability (65 %) due to poor aqueous solubility and extensive first-pass metabolism [15]. The immediate-release and the extended-release forms of doxazosin are used to treat benign prostatic hyperplasia (BPH). The immediate-release tablets are also used to treat high blood pressure. Doxazosin works by blocking the alpha-1a, alpha-1b, and alpha-1d subtypes, which helps to widen blood vessels and relax muscles in the prostate and bladder. The immediate-release formulation can be a second-line agent for managing hypertension in patients with concomitant BPH [16]. The aim of the study is to compare the efficiency of different formulations of DOX in the immediate release form, *i.e.*, nanosuspension, liquid SNEDDS and solid SNEDDS, in improving the release properties of DOX as well as its antihypertensive effect.

## Experimental

### Materials

Doxazosin mesylate, PEG 400, Avicel 101, Aerosil 200, polyvinylpyrrolidone k-30 (PVP K 30), poloxamer 407, microcrystalline cellulose, crospovidone, Ac-Di-Sol, sodium starch glycolate, sodium saccharin, mint flavour, magnesium stearate, talc powder and marketed tablet Dosin® were kindly supplied from Epico Co., Egypt. 10 % glucose solution and oleic acid were purchased from Sigma Chemical Co., St. Louis, MO, USA. All the chemicals used were at analytical grade.

### Preparation of DOX nanosuspension

DOX nanosuspension formula (F) was prepared via emulsion solvent diffusion technique using 533 mg of PVP K-30, 133 mg of Poloxamer-407 and 133 mg of SLS as stabilizers and methanol (100 ml) as a solvent to form a solution of DOX (400 mg). The solution was homogenized (IKA T 25 digital Ultra- Turrax) at 7400 rpm for 7 min, then accelerated to 24,000 rpm for 6 min at room temperature. A probe sonicator (Model GE 50, Scientific Engineering Inc., Woodbridge, Virginia, USA) was then used to sonicate the formed suspension. Finally, 30 ml of distilled water was added to the suspension and stirred using a magnetic stirrer (Type MM5, Poland) for 1 h.

Nanosuspension formed was lyophilized (Heto Power Dry LL1500-Thermo Electron Corporation, USA) for 48 h, at -75 °C at an increasing rate of 5 °C h^-1^ [8], to attain a dry stable form of nanosuspension (F). Glucose solution (10 %) was used as a cryoprotectant.

### Preparation of SNEDDS

According to Ali and Hussein, 2017 [17], the liquid formula of SNEDDS (L) was prepared (10 % oleic acid, 67.5 % Tween 80, 11.25 % PEG 400 and 11.25 % ethanol) in the ratio (oil: Smix (surfactant: co-surfactant mixture) of 1:9), the mixture contains 400 mg DOX.

### Preparation of DOX solid SNEDDS

0.6 g of freshly prepared liquid SNEEDS of DOX (L), equivalent to 400 mg of DOX was dried to the solid form using a mixture of Avicel 101 and Aerosil 200 (600 mg and 15 mg, respectively) in a ratio of (40:1 w/w), their mixing was done in a porcelain mortar.

### Characterization of DOX nanosuspension and SNEDDS

#### Drug content analysis of nanosuspension

10 ml of all freshly made nanosuspension were centrifugated at 10,000 rpm at 25 °C for 10 min using a centrifuge (Model Z 300 K, Hermle Labortechnik Gmbh, Wehingen, Germany). In order to determine the free drug, the supernatant was then analysed at *λ*_max_ 266 nm by UV-VIS spectrophotometer (Genesys 10S UV-VIS, Thermo Spectronic, Waltham, MA, USA). Drug content was calculated using Equation 1 [18].



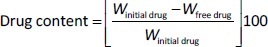

(1)


where, *W*_initial drug_ = weight of the initial drug added and *W*_free drug_ = weight of the free drug in the supernatant.

#### Drug entrapment efficiency of liquid and solid SNEDDS

Precise amounts of liquid and solid SNEDDS equivalent to 2 mg of DOX were diluted using methanol to obtain a concentration of 0.2 mg ml^-1^. Then the solutions were analysed using a UV spectrophotometer at a maximum of 266 nm [15].

#### Measurement of particle/ droplet size and zeta potential

Nanosuspension, liquid and solid SNEDDS were characterized by the measurement of the droplet or particle size and zeta potential by dynamic light scattering (Zetasizer Nano ZS-90, Malvern Instruments, Worcestershire, UK). Distilled water was used for the dilution of each sample 10 times and each measurement was repeated three times [15,19,20]

#### In vitro studies

In vitro dissolution study of pure DOX and nanosuspension (F) and release studies of liquid (L) and solid (S) SNEDDS were performed using Pharma Test dissolution tester type II (Paddle Apparatus, SP6-400 Hamburg, Germany), 500 ml of phosphate buffer pH 6.8 with 1 % tween 80 was used as a release media and maintained at 37±0.2 °C and 100 rpm [12]. In Float-A-Lyzer cellulose ester dialysis tubes (1 mL, molecular weight cut-off of 10 kDa, Spectrum Laboratories, Los Angeles, CA, USA), 8 mg of pure drug and equivalent amounts of tested preparation were added to the dissolution apparatus. Samples (3 ml for each) were withdrawn after (3, 5, 7, 10 and 20 min), then filtered and tested spectrophotometrically at 266 nm. The volume was maintained by using freshly prepared phosphate buffer. The release percent of DOX after 20 min was calculated. The similarity factor (f2) was calculated according to Equation (2).



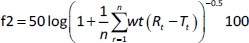

(2)


where, *n* = number of time points, *wt* = optional weighting factor, *r* = the lower limit of summation, *R_t_* / % = amount of drug dissolved of reference product at time *t*, and *T_t_* / % = amount of drug dissolved of test product at that same time point.

#### Transmission electron microscopy

Pure DOX, nanosuspension, liquid, and solid SNEDDS were imaged using transmission electron microscop (TEM) (Jeol jem–2100, Jeol Ltd, Tokyo, Japan) to observe their shape. Each was diluted using distilled water and then added to carbon-coated grids. After this, 2 % phospho-tungstic acid was used for the staining and left for 30 s. Using vacuum dryers, samples were dried by air and imaged by TEM [21].

#### Fourier transform infrared spectroscopy

To detect any interaction occurring between DOX and other ingredients of nanosuspensions and SNEDDS as well as solid SNEDDS, Fourier transform infrared spectroscopy (FTIR) (PerkinElmer 1600 FTIR spectrophotometer, Norwalk, USA) was used. 200 mg of KBr was mixed with each separately, compressed into discs and scanned at a rate of 4 mm s^-1^ in the range of 4000-400 cm^-1^ at 1 cm^-1^ resolution [21].

### Evaluation of the antihypertensive effects of DOX preparations

Adult male albino rats of an average weight ranging from 200 to 250 g were classified into five groups (each group contained five rats). Each group received the treatment mentioned in Table 1 [22] using an oral gavage (Instech Laboratories Inc., Plymouth Meeting, PA, USA). Rats were purchased from an animal breeding centre (Department of Pharmacology, Faculty of Medicine). Hypertension was introduced to the rats according to the Parasuraman and Raveendran method [23]. The mean arterial blood pressure (MAP) was calculated for each rat after treatment with different DOX preparations using Equation 3 [24].

**Table 1.** Classification of the five rat groups

Group no.	Treatment
I	Hypertensive rats treated by market tablet Dosin^®^
II	Hypertensive rats treated by nanosuspension containing 12 mg of DOX nanosuspension (equivalent to 4 mg DOX)
III	Hypertensive rats treated with 1.2 ml of a selected formula of liquid SNNEDDS (equivalent to 4 mg DOX)
IV	Hypertensive rats treated with 1230 mg of a selected formula of solid SNNEDDS (equivalent to 4 mg DOX) redispersed in 1 ml of distilled water.
V	Untreated hypertensive rats (positive control)



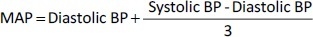

(3)


where, BP is the rat’s blood pressure.

### Statistical analysis

Both in vitro and in vivo data (percent drug release after 3, 5, 7, 10 and 20 min and mean arterial blood pressure of the rats after 0, 15, 30, 60, 90, 120 and 180 min, respectively) were statistically analysed using one-way analysis of variance (ANOVA) using Minitab software, version 17 and the parameters were significant for the *P*< 0.05.

## Results and discussion

### Drug content and entrapment efficiency determination

The drug content of the DOX nanosuspension formulation was 99.99, demonstrating the correctness of the preparation procedure (emulsification solvent diffusion) [25].

Both solid and liquid SNEEDS had a 100 % entrapment efficiency. Tween 80 has a high hydrophilic-lipophilic balance (HLB ) value of 15 and enhances the drug solubility in oleic acid with a subsequent increase in entrapment efficiency [26].

### Measurement of particle/droplet size and zeta potential of DOX nanosuspension, liquid and solid SNEDDS

Both liquid (L) and solid (S) SNEDDS exhibited lower droplet/particle size than DOX nanosuspension (F), as shown in Table 2. This result may be ascribed to the presence of a combination of three stabilizers for nanosuspension preparation in which their amounts should be controlled to obtain particles in the nano range. On the other hand, SNEDDS were prepared in oil: Smix of 1:9 w/w, which was previously reported to produce systems of nanosized droplets (20 to 200 nm) [27]. Nanosuspension's zeta potential is 50.33±4.2 mV owing to its composition (PVP K 30, poloxamer 407 and SLS), which is considered stable in contrast with both L and S possessing lower values and considered electrokinetically unstable. Comparing the zeta potentials of both L and S, it was found that S possesses a higher value than L, possibly due to the presence of an adsorbent mixture. The negative sign of zeta potential is mainly due to the oil content [28], with the adsorbent mixture contributing to the negative sign of zeta potential. Aerosil 200 adsorbs protons on its surface, leading to the ionization of its silanol groups [29], and Avicel 101 also adsorbs sulphate ions on its surface [30].

**Table 2.** Particle size and zeta potential of nanosuspension, liquid SNEDDS and solid SNEDDS.

Formula	Particle size, nm	Zeta potential, mV	Poly-dispersity index
F	385.00±13.00	50.33±4.20	0.44±0.01
L	224.40±15.55	-5.57±0.10	1.00±0.00
S	79.80±14.39	-18.10±0.26	0.47±0.01

### In vitro evaluation of DOX nanosuspension, liquid and solid SNEDDS (F, L and S)

**Figure 1.**
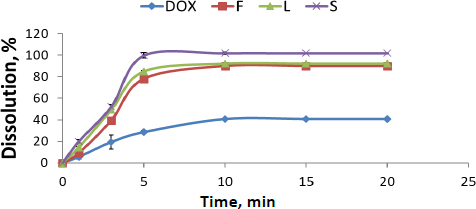
Release profiles of pure DOX, nanosuspension, liquid SNEDDS and solid SNEDDS in phosphate buffer (pH 6.8)

Release percentages of different DOX formulations and their release profiles compared with pure DOX are presented in Figure 1. Pure DOX exhibited poor release properties, about 39±1.00 %. It was 91± 0.43 %, 93± 4.47 % and 100±2.72 % for F, L and S, respectively. It is worth mentioning that the formulation of DOX, either nanosuspension or SNEDDS, had a significant effect on the enhancement of release properties (about a two-fold increase) compared with its pure form. However, solid SNEDDS had a superior effect compared to liquid SNEDDS and DOX nanosuspension (*P* <0.05) due to its smaller particle size. Dissolution rates of F, L and S show a significant difference, confirmed by the similarity factor (f2) between F, L and S, which is 100.

### Characterization of DOX nanosuspension, liquid and solid SNEDDS (F, L and S) using TEM

**Figure 2.**
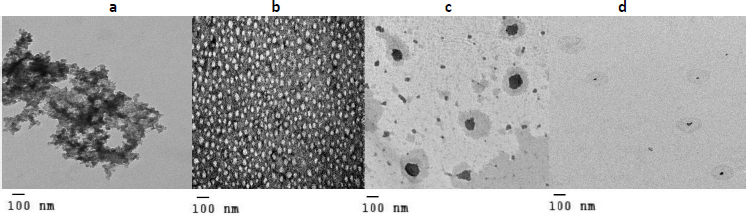
TEM of (a) pure DOX, (b) nanosuspension, (c) liquid SNEDDS, (d) solid SNEDDS.

Characterization results of all DOX formulations revealed significant changes in their shape, as shown in Figure 2b, F appearing as discrete particles [31]. On the other hand, comparing the TEM of pure DOX with the TEM of both L and S (Figures 2a, 2c and 2d, respectively), it was found that pure DOX shape was changed in both c and d with smaller particle size in d, confirming by particle size results [32].

### Characterization of DOX nanxosuspension, liquid and solid SNEDDS using FTIR

**Figure 3.**
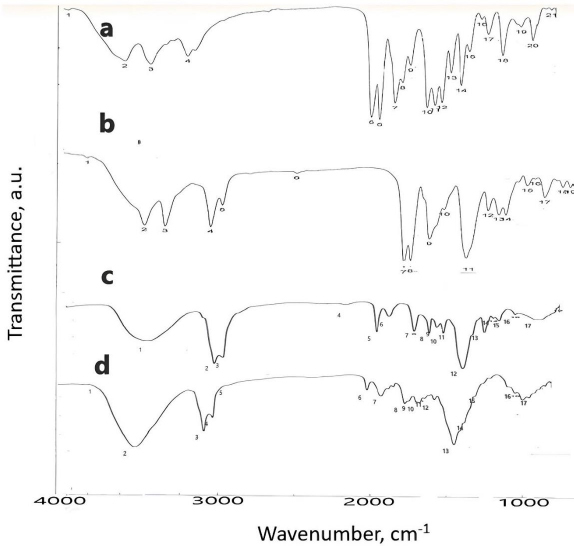
FTIR spectra of (a) pure DOX, (b) nanosuspension, (c) liquid SNEDDS and (d) solid SNEDDS.

Figure 3 shows the IR spectrum of pure DOX, F, L and S. Table 3 shows the characteristic peak of pure DOX (3180 cm^-1^), which reflects the presence of the NH_2_ group, shifted in nanosuspension to 3224 cm^-1^. Another peak in pure DOX at 1651 cm^-1^, reflecting the C=O group, was shifted in nanosuspension to 1642 cm^-1^. The peak at position 1591 cm^-1^, corresponding to the C=N group, was shifted in nanosuspension to 1600 cm^-1^.

**Table 3.** Functional groups of FTIR spectra

Group	Wavenumber, cm ^-1^			
	Pure DOX	F	L	S
NH_2_	3180	3224	-	-
C=O	164	1642	-	-
C-O	1039	-	1248	1248
C = N	1591	1600	1644	1631
C=C	1490	-	1460	1460

Regarding the IR spectra of liquid and solid SNEDDS, the disappearance of the characteristic peaks of DOX reflecting NH_2_ group was observed, suggesting the protonation of the NH_2_ group of DOX forming ion pair with ionized -COOH group of oleic acid. The peak of the C=N group at 1651 cm^-1^ was shifted to 1644 and 1631 cm^-1^ in liquid and solid SNEDDS, respectively. Another peak at 1039 cm^-1^ corresponding to the C-O group was shifted to 1248 cm^-1^ in both liquid and solid SNEDDS. The final peak for the C=C group of DOX appearing in 1490 cm^-1^ was shifted to 1460 cm^-1^ in both liquid and solid SNEDDS. Those changes confirm the presence of liquid and solid SNEDDS, as illustrated previously [20]. The particle size of solid SNEDDS is less than the droplet size of liquid SNEDDS due to the formation of hydrogen bonding between the SNEDDS and -OH group of the adsorbent mixture (Aerosil 200 and Avicel 101) [20].

### In vivo evaluation of DOX nanosuspension, liquid and solid SNEDDS (F, L and S)

**Figure 4.**
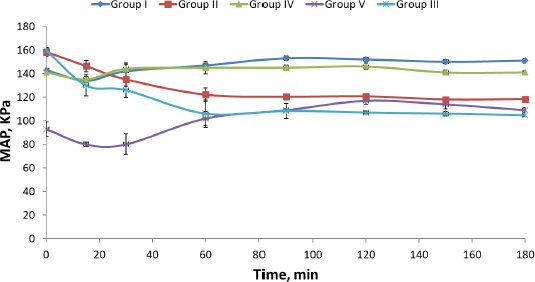
MAP of five rat groups.

Induction of hypertension in rats resulted in a highly significant (*P* = 0.00) increase in their mean arterial blood pressure (MAP). Similar results were reported by Wei *et al.* [33]. Comparing the effectiveness of DOX lyophilized nanosuspension (Group II) with market tablet Dosin® (Group I), it is clear that there was a significant (*P* = 0.016) difference in the MAP of rats of both groups. Also, on comparing rat groups treated with either market tablet Dosin®, L or S (Groups I, III and IV respectively), they showed significant (*P* = 0.00) reduction in MAP compared to the hypertensive non-treated rats (Group V) (Table 4 and Figure 4).

**Table 4.** MAP of the five rat groups (*n* = 5; mean ± SD).

Time, min	MAP, kPA				
	Group I	Group II	Group III	Group IV	Group V*
0	143.66±083	165.33±2.00	158.33±3.51	160.00±2.00	141.00±2.45
15	134.33±1.90	146.66±2.03	146.33±4.72	130.00±9.00	135.33±2.54
30	142.00±6.83	132.00±7.17	135.00±5.77	126.00±6.24	144.33±3.39
60	147.00±1.84	131.66±8.00	122.00±6.00	106.00±11.53	145.33±5.52
90	153.33±2.00	126.33±1.16	120.33±1.52	108.33±6.50	145.00±2.21
120	152.00±1.53	123.00±5.02	120.66±0.57	107.00±1.42	146.00±2.87
150	150.00±1.67	123.33±3.00	118.00±1.73	106.00±2.00	141.66±2.03
180	151.66±2.03	123.00±1.88	118.33±2.08	104.66±1.15	141.66±1.07

*significantly high blood pressure

Dosin® was able to decrease the MAP of hypertensive rats to normal values (Group I) for only 15 min, followed by a re-increase in MAP. On the contrary, F (DOX lyophilized nanosuspension), L and S showed a decrease in MAP of hypertensive rats (Group II, III and IV) to normal values for more than 180 min. Liquid and solid SNEDDS showed a higher decrease in MAP than the nanosuspension owing to the use of Tween 80 (surfactant), which has a high HLB value and creates micelle structures that entrap DOX and improve its solubility. However, the effect of solid SNEDDS on MAP reduction was superior (*P* <0.05) to both liquid SNEDDS and lyophilized nanosuspension. There are statistically significant differences between the MAP of rats in the untreated control group (group V) and the MAP of rats in groups (II, III and IV) as shown in Table 4. This result may be explained on the basis of the difference in particle size between the three formulations.

## Conclusions

This study demonstrated the effectiveness of applied techniques in the preparation of the nano formula of doxazosin mesylate. From the above results, it is clear that the incorporation of DOX in nano-suspension and SEDDS in liquid and solid form leads to an increase in surface area, drug’s solubility and release rate of DOX compared to the pure drug. Incorporation of DOX in solid SNEDDS, liquid SNEDDS and nanosuspension led to a significant reduction in MAP of hypertensive rats for more than 180 min when compared with the marketed tablet (Dosin®).
